# Unveiling Influence of Dielectric Losses on the Localized Surface Plasmon Resonance in (Al,Ga)As:Sb Metamaterials

**DOI:** 10.3390/nano14020167

**Published:** 2024-01-12

**Authors:** Vitalii I. Ushanov, Sergey V. Eremeev, Vyacheslav M. Silkin, Vladimir V. Chaldyshev

**Affiliations:** 1 Ioffe Institute, 26 Politekhnicheskaya Str., 194021 Saint Petersburg, Russia; ushanovvi@mail.ioffe.ru; 2 Institute of Strength Physics and Materials Science, Siberian Branch, Russian Academy of Sciences, 634055 Tomsk, Russia; eremeev@ispms.tsc.ru; 3 Departamento de Polímeros y Materiales Avanzados: Física, Química y Tecnología, Facultad de Ciencias Químicas, Universidad del País Vasco (UPV-EHU), Apdo. 1072, E-20080 San Sebastián, Spain; 4 Donostia International Physics Center (DIPC), Paseo de Manuel Lardizabal 4, E-20018 San Sebastián, Spain; 5 Ikerbasque, Basque Foundation for Science, E-48009 Bilbao, Spain

**Keywords:** metal–semiconductor metamaterials, nanoparticles, dielectric function, optical absorption, plasmon resonance

## Abstract

We perform numerical modeling of the optical absorption spectra of metamaterials composed of systems of semimetal antimony nanoparticles embedded into ${\mathrm{Al}}_{x}$ ${\mathrm{Ga}}_{1-x}$ As semiconductor matrices. We reveal a localized surface plasmon resonance (LSPR) in these metamaterials, which results in a strong optical extinction band below, near, or above the direct band gap of the semiconductor matrices, depending on the chemical composition of the solid solutions. We elucidate the role of dielectric losses in ${\mathrm{Al}}_{x}$ ${\mathrm{Ga}}_{1-x}$ As, which impact the LSPR and cause non-plasmonic optical absorption. It appears that even a dilute system of plasmonic Sb nanoinclusions can substantially change the optical absorption spectra of the medium.

## 1. Introduction

The interaction of light with metallic nanoparticles can enable the localization and amplification of optical fields on subwavelength scales. These phenomena are induced by the interaction of the light wave with the inherent localized excitations of the electron plasma within the nanoparticles. If an array of such nanoparticles is formed in a dielectric or semiconductor environment, its dielectric and optical properties are significantly modified. In such a metal-dielectric (metal–semiconductor) composite metamaterial, localized surface plasmon resonances (LSPR) arise, the characteristics of which depend on both the properties of the nanoparticle ensemble and the properties of the matrix [1]. Such a medium can acquire unusual linear and nonlinear optical properties [2,3,4,5,6,7,8,9]. An example of a nonlinear response is saturable absorbers, which operate in the spectral region of LSPR excitation [10]. In comparison, for instance, with a system of quantum dots in semiconductors, the system of metallic nanoparticles provides significantly greater efficiency in interaction with light and much shorter relaxation times of optical properties [11].

Metal–semiconductor metamaterials can be integrated into devices with semiconductor lasers, light-emitting diodes, and other optoelectronic components if the materials and fabrication technologies are consistent. Unfortunately, such integration often proves to be impossible as the technology for forming nanoparticles of common plasmonic metals, silver or gold, is incompatible with the technology of epitaxial growth of III-V semiconductors, which are widely used in optoelectronics [12]. In this case, the system of plasmonic nanoparticles can be obtained after the growth of the semiconductor structure on its surface [13,14].

A unique opportunity to form arrays of plasmonic nanoparticles in epitaxial layers of GaAs and AlGaAs appears when these materials are grown by molecular beam epitaxy (MBE) at low temperature (LT) under a strong flux of As atoms compared to the flux of Ga atoms [15,16]. Epitaxial layers of LT-GaAs grown in this way possess high crystalline quality but contain a high concentration of excess superstoichiometric arsenic predominantly in the form of anti-site defects ${\mathrm{As}}_{\mathrm{Ga}}$ [17,18]. The concentration of these defects can significantly exceed equilibrium values and reach up to 2 at.%. Subsequent high-temperature annealing leads to the activation of diffusion processes and the formation of nanometer-sized precipitates of semimetallic As due to self-organization processes in a metastable environment [16,19]. The semiconductor medium meanwhile retains the high crystalline quality corresponding to the standards of growth and formation of epitaxial structures.

The presence of an array of arsenic nanoparticles does not lead to significant changes in the optical properties in the near-infrared transparency window of the GaAs matrix [16,20]. However, in the case of the LT MBE layers of the ${\mathrm{GaAs}}_{1-y}$ ${\mathrm{Sb}}_{y}$ and ${\mathrm{Al}}_{x}$ ${\mathrm{Ga}}_{1-x}$ ${\mathrm{As}}_{1-y}$ ${\mathrm{Sb}}_{y}$ (where x≈0.3 , y≈0.03 ) solid solutions, absorption associated with the AsSb nanoinclusions enriched with antimony was detected in optical spectra [21,22]. The magnitude of this absorption increased with photon energy up to the fundamental absorption edge of the matrix. Such an absorption was interpreted as the LSPR in the AsSb nanoparticle system. To reliably determine the parameters of this resonance, the transparency window of the ${\mathrm{Al}}_{x}$ ${\mathrm{Ga}}_{1-x}$ ${\mathrm{As}}_{1-y}$ ${\mathrm{Sb}}_{y}$ semiconductor matrix was expanded by increasing the aluminum concentration *x* to 0.6 [23,24]. Optical studies of this metamaterial with a developed system of Sb-rich nanoinclusions revealed a plasmonic absorption band in the spectral region λ>600 nm near the absorption edge caused by direct interband transitions in the ${\mathrm{Al}}_{x}$ ${\mathrm{Ga}}_{1-x}$ ${\mathrm{As}}_{1-y}$ ${\mathrm{Sb}}_{y}$ matrix [23,24].

The plasmonic nature of the observed optical absorption was confirmed [25] by modeling in terms of Mie theory [26]. The modeling utilized the complex dielectric functions of the AsSb alloy of various compositions calculated by the density functional theory. The Adachi model was used to describe the dielectric properties of the ${\mathrm{Al}}_{x}$ ${\mathrm{Ga}}_{1-x}$ As matrix with an aluminum content x=0.6 [27]. In this model, the dielectric function was frequency-dependent and real. The calculations showed that for the observation of LSPR in the AsSb- ${\mathrm{Al}}_{x}$ ${\mathrm{Ga}}_{1-x}$ As system, it is necessary that the chemical composition of the nanoparticles be close to a pure Sb. As the arsenic content in the nanoparticles increases, the LSPR energy shifts to 3 eV, i.e., into the spectral region where the ${\mathrm{Al}}_{x}$ ${\mathrm{Ga}}_{1-x}$ As matrix is optically opaque, even at the maximum Al concentration x=1 .

The assumption of a small imaginary part of the dielectric permittivity of the semiconductor is obviously justified in the transparency region of the material, where light absorption is low. However, near the fundamental absorption edge caused by direct interband transitions of electrons, the imaginary part of the dielectric permittivity significantly increases. Accounting for the dielectric losses in the ${\mathrm{Al}}_{x}$ ${\mathrm{Ga}}_{1-x}$ As matrix can substantially change the LSPR parameters and the optical properties of the metamaterial in this spectral region. In fact, the dipole polarizability of a small spheric nanoparticle with radius r≪λ ( λ is the light wavelength) and dielectric permittivity ${\tilde{\varepsilon }}_{\mathrm{sph}}$ embedded in a medium with dielectric permittivity ${\tilde{\varepsilon }}_{m}$ is [1]

(1) $$\chi =4\pi r^{3}\frac{{\tilde{\varepsilon }}_{\mathrm{sph}}-{\tilde{\varepsilon }}_{m}}{{\tilde{\varepsilon }}_{\mathrm{sph}}+2{\tilde{\varepsilon }}_{m}},$$

where both ${\tilde{\varepsilon }}_{\mathrm{sph}}$ and ${\tilde{\varepsilon }}_{m}$ are frequency-dependent complex functions. So, the dielectric losses in the ${\mathrm{Al}}_{x}$ ${\mathrm{Ga}}_{1-x}$ As matrix described by the imaginary part of ${\tilde{\varepsilon }}_{m}$ can affect the parameters of LSPR. In addition, the complex dielectric function ${\tilde{\varepsilon }}_{m}$ implies a non-plasmonic optical absorption in the semiconductor matrix itself. This absorption becomes very strong for the photons with energy exceeding the fundamental absorption edge caused by direct band-to-band transitions. At the high-energy end of the optical spectra, the absorption in the matrix should govern the optical properties of the metamaterial. So, both phenomena related to dielectric losses in the semiconductor matrix should be taken into account to make the theoretical predictions suitable for direct meaningful comparison with experimental data.

A model representation of the dielectric function of the ${\mathrm{Al}}_{x}$ ${\mathrm{Ga}}_{1-x}$ As semiconductor solid solutions considering dielectric losses was developed by Djurišić et al. [28] (hereafter referred to briefly as the Djurišić model). In this model, the dielectric function is complex. The description of the real part of the dielectric permittivity in the Djurišić model is similar to that in the Adachi model in the energy range where ${\mathrm{Al}}_{x}$ ${\mathrm{Ga}}_{1-x}$ As is optically transparent. The difference in the two models makes it possible to examine the influence of dielectric losses in the ${\mathrm{Al}}_{x}$ ${\mathrm{Ga}}_{1-x}$ As matrix on the LSPR parameters in the system of Sb plasmonic nanoparticles and, consequently, on the optical properties of such a metamaterial.

**Figure 1:**
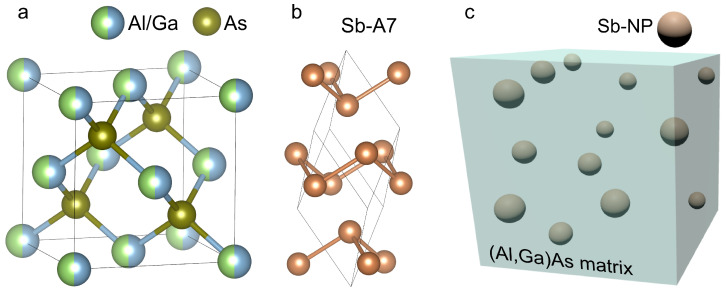
Atomic structures of the zincblende (Al,Ga)As (**a**), A7 phase of Sb (**b**), and schematic presentation of the (Al,Ga)As:Sb metamaterial (**c**).

In this study, keeping in mind the experimental data [20,21,22,23,24], we employ the Mie theory to calculate the optical absorption spectra of metamaterials based on crystalline ${\mathrm{Al}}_{x}$ ${\mathrm{Ga}}_{1-x}$ As semiconductor matrices with an embedded system of Sb nanoparticles (see Figure 1). The dielectric properties of antimony have been described using our data from ab initio calculations reported in Ref. [25]. The dielectric properties of the ${\mathrm{Al}}_{x}$ ${\mathrm{Ga}}_{1-x}$ As solid solutions have been described by using the two models mentioned above. We reveal changes in the LSPR optical absorption due to the dielectric losses in the ${\mathrm{Al}}_{x}$ ${\mathrm{Ga}}_{1-x}$ As matrix. We compare the optical extinction due to LSPR and due to direct interband transitions in the matrix. We show that the former prevails near and below the direct bang gap in the semiconductor matrix, whereas the latter dominates in the high-energy part of the optical spectra where several direct electronic band-to-band transitions are allowed.

## 2. Dielectric Functions of ${\mathrm{Al}}_{x}$ ${\mathrm{Ga}}_{1-x}$ As and Sb

${\mathrm{Al}}_{x}$ ${\mathrm{Ga}}_{1-x}$ As semiconductor solid solutions possess a zincblende structure with atomic arrangement illustrated in Figure 1a. The Adachi model [27] is widely used to reliably describe the optical properties of the ${\mathrm{Al}}_{x}$ ${\mathrm{Ga}}_{1-x}$ As material with a different Al composition *x*. The real part, $\varepsilon_{1}$ , of the dielectric function below the direct bandgap $E_{0}$ reads

(2) $$\varepsilon_{1}\left(\omega \right)=A\left\{f\left(\chi \right)+\frac{1}{2}{\left[\frac{E_{0}}{E_{0}+\Delta_{0}}\right]}^{\frac{3}{2}}f\left(\chi_{\mathrm{so}}\right)\right\}+B,$$

where

(3) $$f\left(\chi \right)=\chi^{-2}\left[2-{\left(1+\chi \right)}^{\frac{1}{2}}-{\left(1-\chi \right)}^{\frac{1}{2}}\right],$$

(4) $$\chi_{\mathrm{so}}=\frac{\hbar \omega }{\left(E_{0}+\Delta_{0}\right)},$$

(5) $$\chi =\frac{\hbar \omega }{E_{0}}.$$

Here, ℏω is the photon energy, $\Delta_{0}$ is the spin–orbit splitting of the valence band in ${\mathrm{Al}}_{x}$ ${\mathrm{Ga}}_{1-x}$ As, *A* and *B* are parameters. The terms in curly brackets in Equation (2) represent contributions from free electron–hole pairs, as well as excitons associated with the bands $E_{0}$ and $E_{0}+\Delta_{0}$ . The parameter *B* determines the background dielectric permittivity provided by contributions from higher bands $E_{1}$ , $E_{1}+\Delta_{1}$ , and $E_{2}$ . The dependencies of parameters *A* and *B* on the aluminum concentration, *x*, are as follows:

(6) A=6.3+19.0x,

(7) B=9.4−10.2x.

**Figure 2:**
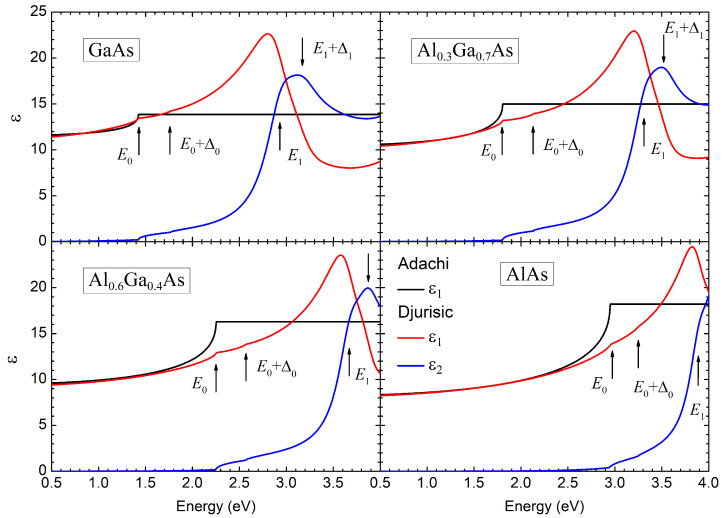
The real ( $\varepsilon_{1}$ ) and imaginary ( $\varepsilon_{2}$ ) parts of the dielectric permittivities for GaAs, AlAs, and ${\mathrm{Al}}_{x}$ ${\mathrm{Ga}}_{1-x}$ As solid solutions with aluminum content x=0.3 and 0.6 according to Adachi [27] and Djurišić [28] models. Arrows indicate the energies of the major critical points.

For photon energies above $E_{0}$ , the real part of the dielectric function is assumed to be constant. The dispersion curves of $\varepsilon_{1}$ for GaAs, AlAs, and ${\mathrm{Al}}_{x}$ ${\mathrm{Ga}}_{1-x}$ As solid solutions with aluminum content x=0.3 and 0.6 are plotted in Figure 2 by black lines. Arrows indicate the energy of the optical transitions in the critical points of the Brillouin zone.

In the Djurišić model [28], Adachi’s approach was extended by considering additional contributions from the critical points $E_{o}^{\prime }$ , $E_{2}\left(X\right)$ , and $E_{2}\left(\Sigma \right)$ and by including damped Wannier excitons. The final expression for the complex dielectric function is as follows:

(8) $${\tilde{\varepsilon }}_{m}\left(\omega \right)=\varepsilon^{I}\left(\omega \right)+\varepsilon^{\mathrm{II}}\left(\omega \right)+\varepsilon^{\mathrm{III}}\left(\omega \right)+\varepsilon^{\mathrm{IV}}\left(\omega \right)+\varepsilon_{\infty }.$$

The last component, $\varepsilon_{\infty }$ , corresponds to the high-frequency dielectric permittivity. The first term, $\varepsilon^{I}\left(\omega \right)$ , represents transitions at three-dimensional critical points $E_{0}$ and $E_{0}+\Delta_{0}$ under the parabolic band assumption. The second component accounts for transitions at two-dimensional critical points $M_{0}$ , $E_{1}$ , and $E_{1}+\Delta_{1}$ . The third component is due to the Wannier excitons (the discrete series of excitonic lines for $E_{1}$ and $E_{1}+\Delta_{1}$ ). Features of the spectrum related to the transitions $E_{o}^{\prime }$ , $E_{2}\left(X\right)$ , and $E_{2}\left(\Sigma \right)$ , which are described in the model of damped harmonic oscillators, are defined by the fourth term.

The energies of the critical points $E_{0}$ , $E_{0}+\Delta_{0}$ , $E_{1}$ and $E_{1}+\Delta_{1}$ were determined by a cubic approximation with respect to *x* derived in Ref. [29]

(9) $$E_{i}\left({\mathrm{Al}}_{x}{\mathrm{Ga}}_{1-x}\mathrm{As}\right)=E_{i}\left(\mathrm{GaAs}\right)+\left[E_{i}\left(\mathrm{AlAs}\right)-E_{i}\left(\mathrm{GaAs}\right)\right]x+\left(c_{0}+c_{1}x\right)x\left(1-x\right).$$

The values of $E_{i}\left(\mathrm{GaAs}\right)$ , $E_{i}\left(\mathrm{AlAs}\right)$ , $c_{0}$ , and $c_{1}$ for the critical points are provided in Table 1. To describe the composition dependencies of the remaining model parameters, linear interpolation was used. The complete list of parameters, as well as a detailed description of the model, can be found in Ref. [28].

The dispersion of the real ( $\varepsilon_{1}$ ) and imaginary ( $\varepsilon_{2}$ ) parts of the dielectric function are represented in Figure 2 for the binary GaAs and AlAs compounds and their solid solutions ${\mathrm{Al}}_{x}$ ${\mathrm{Ga}}_{1-x}$ As with aluminum contents x=0.3 and 0.6 . In the optical transparency region of the semiconductor ( $\hbar \omega <E_{0}$ ), the real parts of the dielectric function calculated within the two models are close to each other. Significant differences in $\varepsilon_{1}$ occur near the edge of the direct bandgap ( $E_{0}$ ), where the Djurišić model produces a smoother and weaker spectral feature. With a further increase in energy, the value of $\varepsilon_{1}$ according to the Djurišić model increases due to contributions from the critical points $E_{1}$ and $E_{1}+\Delta_{1}$ . In the Adachi model, this spectral region is not described and $\varepsilon_{1}$ is assumed to be a constant in calculations and equal to the value at the bandgap edge $\varepsilon_{1}\left(E_{0}\right)$ .

The imaginary part of the dielectric function in the Djurišić model is determined by transitions near the critical points in the band structure. The most pronounced peak in the $\varepsilon_{2}$ dispersion curve corresponds to the transitions at $E_{1}$ and $E_{1}+\Delta_{1}$ . Smooth steps on the $\varepsilon_{2}$ spectra near photon energy of 1.4 eV for GaAs, 1.8 eV for ${\mathrm{Al}}_{0.3}$ ${\mathrm{Ga}}_{0.7}$ As, 2.2 eV for ${\mathrm{Al}}_{0.6}$ ${\mathrm{Ga}}_{0.4}$ As, and 3.0 eV for AlAs correspond to the width of the direct bandgap $E_{0}$ . It is worth noting that the presence of indirect interband transitions does not contribute significantly and does not create noticeable spectral features for the real and imaginary parts of the dielectric function.

In accordance with the Kramers–Kronig relations, the dielectric function in the Djurišić model remains complex even in the energy region below the bandgap. The corresponding dielectric losses are described by exponentially decaying tails on the $\varepsilon_{2}$ spectra.

The complex dielectric function of Sb was computed using the time-dependent density functional theory [31,32] employing our band structure and dielectric function calculation tools [33,34]. We considered the common crystalline phase of antimony packed in A7 rhombohedral lattice [35]. The corresponding atomic arrangement is illustrated in Figure 1b. Calculation details and the obtained band structure and dielectric functions are described in Ref. [25]. In accordance with the lattice symmetry, the dielectric function of Sb demonstrated significant anisotropy. Thus, its real part becomes negative at energies above 1.0 eV (1.85 eV) for the in-plane (out-of-plane) polarization [25]. On the other hand, on the low-energy side, the semi-metal behavior of Sb is reflected in the presence of a Drude peak in the imaginary part and the negative values of $\varepsilon_{1}$ at low energies. The latter becomes positive at energies exceeding 0.25 eV (0.30 eV) for the in-plane (out-of-plane) momentum direction due to interband transitions. A prominent peak in $\varepsilon_{2}$ at 1.8 eV originates from the interband transitions involving the parallel sections in the occupied and unoccupied energy bands in the vicinity of the H point, especially along the H-L symmetry direction. Additionally, some contributions are provided by the short parallel regions along the Γ -M and Γ -K symmetry directions.

**Figure 3:**
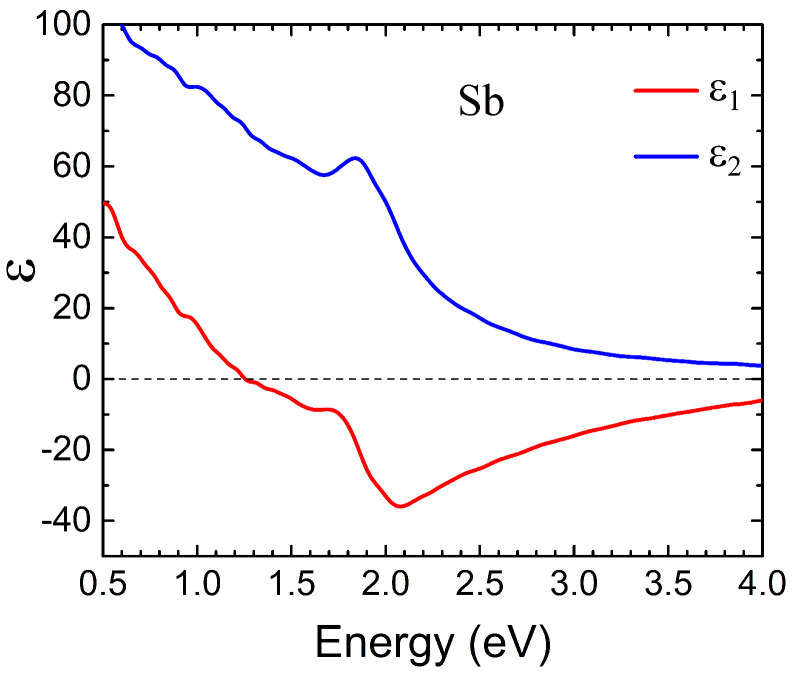
The real ( $\varepsilon_{1}$ ) and imaginary ( $\varepsilon_{2}$ ) parts of the averaged Sb dielectric function calculated employing the *ab initio* data of Ref. [25].

For LSPR calculations, the dielectric properties were averaged over all three crystallographic directions. The resulting averaged real and imaginary parts of the dielectric permittivity are shown in Figure 3. It is evident that in this case, the real part of the dielectric permittivity is negative in the energy region above 1.2 eV, which creates the possibility for the formation of surface plasmon resonances at the interface with a dielectric media. However, the imaginary part of the dielectric permittivity exceeds 40 in the energy range between 1.2 and 2.1 eV, which should result in a strong damping of any possible resonances. In the case where the dielectric is an ${\mathrm{Al}}_{x}$ ${\mathrm{Ga}}_{1-x}$ As semiconductor solid solution, the LSPR conditions are expected to occur in the energy range of 2.5–2.9 eV [25], where the imaginary part of permittivity gradually reduces from 18 to 10.

## 3. Modeling the Optical Properties of the Sb Nanoparticle Ensemble in ${\mathrm{Al}}_{x}$ ${\mathrm{Ga}}_{1-x}$ As

The numerical modeling of the optical extinction coefficient dispersion in a system of spherical Sb nanoparticles in ${\mathrm{Al}}_{x}$ ${\mathrm{Ga}}_{1-x}$ As was performed using Mie theory [26]. A sketch of the metamaterial structure is presented in Figure 1c. The optical extinction coefficient in a system of metallic spheres in a semiconductor or dielectric medium in the limit of low concentration of the particles is given by the expression

(10) $$\alpha =\frac{3}{4}\frac{f}{\pi r^{3}}C_{\mathrm{ext}},$$

where the filling factor *f* is the volume fraction occupied by the metallic particles with radius *r*, and $C_{\mathrm{ext}}$ is the optical extinction cross-section, defined for a single nanoparticle as follows [26]:

(11) Cext=2πk2∑i=1∞(2i+1)Re(ai+bi).

Here, *k* is the wave number for light in vacuum and $a_{i}$ and $b_{i}$ are the scattering coefficients. Assuming equal magnetic permeabilities of the particle and surrounding matrix, the coefficients are determined as [26]

(12) $$a_{i}=\frac{\tilde{m}\psi_{i}\left(\tilde{m}x\right)\psi_{i}^{\prime }\left(x\right)-\psi_{i}\left(x\right)\psi_{i}^{\prime }\left(\tilde{m}x\right)}{\tilde{m}\psi_{i}\left(\tilde{m}x\right)\xi_{i}^{\prime }\left(x\right)-\xi_{i}\left(x\right)\psi_{i}^{\prime }\left(\tilde{m}x\right)},$$

(13) $$b_{i}=\frac{\psi_{i}\left(\tilde{m}x\right)\psi_{i}^{\prime }\left(x\right)-\tilde{m}\psi_{i}\left(x\right)\psi_{i}^{\prime }\left(\tilde{m}x\right)}{\psi_{i}\left(\tilde{m}x\right)\xi_{i}^{\prime }\left(x\right)-\tilde{m}\xi_{i}\left(x\right)\psi_{i}^{\prime }\left(\tilde{m}x\right)},$$

where ${\tilde{m}}^{2}={\tilde{\varepsilon }}_{\mathrm{sph}}/{\tilde{\varepsilon }}_{m}$ , x=kr , $\psi_{i}\left(\rho \right)$ and $\xi_{i}\left(\rho \right)$ are the Riccati–Bessel functions.

In the case when the nanoparticles are significantly smaller than the wavelength of the radiation, x≪1 , the final expression for the total optical extinction coefficient can be significantly simplified

(14) αext=αm+3fkImε˜sph−ε˜mε˜sph+2ε˜m,

where $\alpha_{m}$ is the optical absorption coefficient in the matrix. It is evident from Equation (14) that in the case of small nanoparticles occupying a fixed total portion of the medium, the absorption magnitude does not depend on the size of the nanoparticles, it is determined solely by the filling factor *f*.

## 4. Results and Discussion

**Figure 4:**
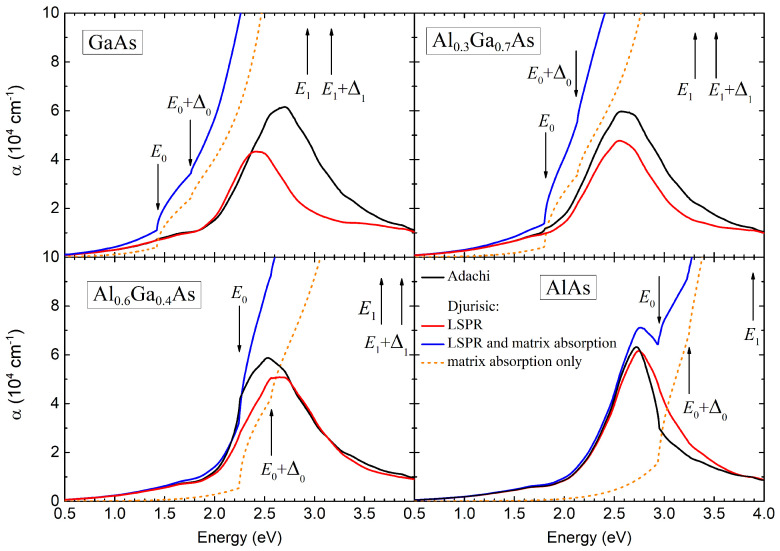
The optical extinction spectra of the compositional metamaterial based on the systems of Sb nanoinclusions in GaAs and AlAs binary compounds and ${\mathrm{Al}}_{x}$ ${\mathrm{Ga}}_{1-x}$ As solid solutions with aluminum contents x=0.3 and 0.6 . The filling factor and nanoparticle radius used in calculations were f=0.016 and 3.1 nm, respectively. Solid black and red lines represent optical extinction due to LSPR, calculated using the Adachi [27] and Djurišić [28] models. The dashed orange curves show absorption in the semiconductor matrix according to the Djurišić model in the absence of Sb nanoparticles. The solid blue curves are the cumulative optical extinction spectra of the metamaterial.

The calculated optical extinction spectra for the ${\mathrm{Al}}_{x}$ ${\mathrm{Ga}}_{1-x}$ As:Sb metamaterial are presented in Figure 4 for four compositions x=0,0.3,0.6,1.0 of the semiconductor solid solution. The Sb nanoparticle radius is taken as 3.1 nm, and the filling factor f=0.016 . These values correspond to the results of the experimental structural studies of Sb-enriched nanoinclusions formed in GaAs(Sb), ${\mathrm{Al}}_{0.3}$ ${\mathrm{Ga}}_{0.7}$ As(Sb), and ${\mathrm{Al}}_{0.6}$ ${\mathrm{Ga}}_{0.4}$ As(Sb) [23,24].

It is evident from Figure 4 that the Sb nanoparticle system embedded in the ${\mathrm{Al}}_{x}$ ${\mathrm{Ga}}_{1-x}$ As matrix forms a band of resonant light absorption in the optical spectra, which is due to LSPR. Calculations using the Adachi model (black curves) give the peak position $\hbar \omega_{\mathrm{LSPR}}$ at 2.67, 2.60, 2.54, and 2.72 eV for the matrix compositions x=0 , 0.3 , 0.6 , and 1, respectively. Accounting for the dispersion and complex nature of the dielectric permittivity of the semiconductor medium via Djurišić model leads to changes in the spectrum of plasmonic light absorption. Red curves in Figure 4 show that the LSPR peak shifts to the photon energy of 2.43 eV for the GaAs matrix, to 2.55 eV for ${\mathrm{Al}}_{0.3}$ ${\mathrm{Ga}}_{0.7}$ As, to 2.63 eV for ${\mathrm{Al}}_{0.6}$ ${\mathrm{Ga}}_{0.4}$ As, and to 2.74 eV for AlAs. These values correspond to the fulfillment of the condition for LSPR: Re(ε˜sph+2ε˜m)=0 .

Arrows in Figure 4 indicate major critical points of the optical transitions in the semiconductor matrix. It is clear that for the GaAs and ${\mathrm{Al}}_{0.3}$ ${\mathrm{Ga}}_{0.7}$ As matrices, the plasmon absorption band is situated deeply within the fundamental absorption band of the semiconductor materials. In the case of the ${\mathrm{Al}}_{0.6}$ ${\mathrm{Ga}}_{0.4}$ As matrix, the LSPR energy is near the $E_{0}+\Delta_{0}$ critical point of interband transitions. In the case of the AlAs matrix, the LSPR is manifested as a peak in the spectral region ( $E_{g}^{X}<\hbar \omega_{\mathrm{LSPR}}<E_{0}$ ) where the optical absorption in the semiconductor is determined by indirect interband transitions.

Taking into consideration the imaginary part of the dielectric permittivity of the matrix leads to a reduction in the magnitude of the LSPR optical extinction peak (compare red and black curves in Figure 4). This additional damping of the LSPR is rather negligible in the case of the AlAs matrix when the LSPR energy is smaller than the direct band gap, $\hbar \omega <E_{0}$ . The reduction in the magnitude of the LSPR peak becomes noticeable for ${\mathrm{Al}}_{0.6}$ ${\mathrm{Ga}}_{0.4}$ As; ${\mathrm{Al}}_{0.3}$ ${\mathrm{Ga}}_{0.7}$ As; and, especially, for the GaAs matrix, where the energies of critical points $E_{0}$ , $E_{0}+\Delta_{0}$ and others are the lowest. The dielectric losses in the GaAs matrix strongly reduce the high-energy part of the resonant band, whereas the low-energy side of the band ( $\hbar \omega <\hbar \omega_{\mathrm{LSPR}}$ ) remains almost unchanged even when $\hbar \omega >E_{0}$ . It is also interesting to note that additional dielectric losses do not result in the broadening of the LSPR extinction peak.

Dielectric losses in the semiconductor matrix lead not only to changes in the parameters of the LSPR but also result in additional optical absorption, $\alpha_{m}$ , within the medium, which is unrelated to LSPR. Such absorption is, of course, absent when using the Adachi model, where the dielectric permittivity is considered real. The dashed orange lines in Figure 4 represent the optical absorption spectra of GaAs, ${\mathrm{Al}}_{0.3}$ ${\mathrm{Ga}}_{0.7}$ As, ${\mathrm{Al}}_{0.6}$ ${\mathrm{Ga}}_{0.4}$ As, and AlAs matrices, calculated with the complex dielectric function prescribed by the Djurišić model. It is evident that the absorption coefficient $\alpha_{m}$ is relatively small and quickly decreases when the photon energy is below the direct band gap $E_{0}$ . When the photon energy exceeds the threshold for direct interband transitions $\hbar \omega >E_{0}$ , the absorption coefficient sharply increases. For higher photon energy $\hbar \omega >E_{0}+\Delta_{0}$ and then $\hbar \omega >E_{1}$ , the additional interband transitions make the semiconductor matrix more and more opaque with an absorption coefficient eventually exceeding $\alpha_{m}>1\times 10^{5}$ ${\mathrm{cm}}^{-1}$ .

The spectra of total optical extinction is plotted in Figure 4 by blue curves. For any chemical composition of the ${\mathrm{Al}}_{x}$ ${\mathrm{Ga}}_{1-x}$ As matrices ( 0≥x≥1 ), the energy of the LSPR $\hbar \omega_{\mathrm{LSPR}}$ provided by the systems of embedded Sb nanoparticles is less than $E_{1}$ . Therefore, the optical absorption by plasmonic nanoparticles makes a substantial or dominating contribution to the total absorption in a wide spectral range near and below $\hbar \omega_{\mathrm{LSPR}}$ . For instance, even in the case of GaAs matrix, when $E_{0}$ , $E_{1}$ and other critical points have the lowest energies, the LSPR absorption at $\hbar \omega_{\mathrm{LSPR}}=2.43$ eV equals $4\times 10^{4}$ ${\mathrm{cm}}^{-1}$ , whereas the absorption in the matrix is as high as $9\times 10^{4}$ ${\mathrm{cm}}^{-1}$ .

In the case of ${\mathrm{Al}}_{0.6}$ ${\mathrm{Ga}}_{0.4}$ As matrix the LSPR energy is near the $E_{0}+\Delta_{0}$ critical point of interband transitions in the semiconductor. At this energy, the two mechanisms make approximately equal contributions to the total absorption. Both mechanisms cause lower absorption for the photon energy decreasing to $E_{0}$ . This decrease is sharper for the direct band-to-band transition in the matrix than for the LSPR. So, even when the LSPR energy $\hbar \omega_{LSPR}$ is substantially above the direct band gap $E_{0}$ in the semiconductor matrix, a large width of the LSPR provides a strong absorption for photons with energy near $E_{0}$ . In the case of the ${\mathrm{Al}}_{0.6}$ ${\mathrm{Ga}}_{0.4}$ As matrix, the absorption coefficient due to LSPR at $\hbar \omega =E_{0}$ is about $3\times 10^{4}$ ${\mathrm{cm}}^{-1}$ .

The LSPR absorption substantially prevails over the optical absorption in the semiconductor matrix at and below $E_{0}$ . In this spectral range, the absorption in the semiconductors is governed by exponentially decaying “tails” below the direct band gap and by indirect optical transitions in the case of ${\mathrm{Al}}_{x}$ ${\mathrm{Ga}}_{1-x}$ As matrices with x≥0.41 [30]. Such dielectric losses are included in the Djurišić model, which prescribes substantial tails in the absorption coefficient as shown in Figure 4.

For the AlAs matrix, the LSPR optical extinction peak occurs for photons with energy below the edge of fundamental absorption $\hbar \omega_{\mathrm{LSPR}}<E_{0}$ . The absorption coefficient reaches a peak value of $6\times 10^{4}$ ${\mathrm{cm}}^{-1}$ at ℏω=2.74 eV. The absorption in the semiconductor matrix in this case is significantly lower than the plasmonic one. Notably, such high values of the optical extinction coefficient are achieved by a relatively small amount of incorporated Sb nanoparticles, which in total occupy only 1.6% of the entire volume of the medium. Due to the small size of the nanoparticles compared to the light wavelength, the plasmonic response is predominantly dipolar.

It is interesting to consider the absorption in the metamaterials based on systems of Sb plasmonic nanoparticles embedded into GaAs, ${\mathrm{Al}}_{0.3}$ ${\mathrm{Ga}}_{0.7}$ As, and ${\mathrm{Al}}_{0.6}$ ${\mathrm{Ga}}_{0.4}$ As matrices for photons with energy below $\hbar \omega <E_{0}<\hbar \omega_{\mathrm{LSPR}}$ . It is evident from Figure 4 that the plasmonic contribution to the optical extinction coefficient predominates over the contribution provided by the semiconductor matrix even though we consider a dilute system of Sb nanoparticles and the LSPR maximum occurs at much higher energies than the direct band gap. This fact indicated a high oscillator strength and a large homogeneous spectral width of the LSPR.

It is important to note that the results of the above-described modeling of the optical spectra are fully consistent with the existing experimental data obtained for metamaterials with Sb-rich AsSb nanoinclusions built in GaAs, ${\mathrm{Al}}_{0.3}$ ${\mathrm{Ga}}_{0.7}$ As, and ${\mathrm{Al}}_{0.6}$ ${\mathrm{Ga}}_{0.4}$ As semiconductor matrices [21,22,23,24].

## 5. Conclusions

Our study of the optical properties of metamaterials based on the Sb plasmonic nanoparticle system in the ${\mathrm{Al}}_{x}$ ${\mathrm{Ga}}_{1-x}$ As matrix shows that the dielectric losses and the dispersion of the dielectric permittivity in the matrix, along with the dielectric losses and the dispersion of the dielectric permittivity of the nanoparticle material, noticeably affect the parameters of LSPR. This influence is strongest in the region of higher energies $\hbar \omega \geq E_{1}$ (see Table 1) when the dielectric losses in the semiconductor become comparable to the dielectric losses in the semimetal. In addition, in this case, LSPR occurs with the background of strong interband absorption in the semiconductor matrix. If the volume fraction of the medium occupied by the nanoinclusions is as small as 1.6%, the total optical absorption in the metamaterial within the high-energy spectral range is mostly governed by the direct band-to-band transitions in the semiconductor matrix. Increasing the filling factor should, of course, lead to an increase in the magnitude of LSPR; however, achieving large filling factor values f>0.02 via the self-organization technology based on LT MBE is extremely difficult [15,16].

The dielectric losses in the matrix have little effect on the LSPR parameters when the resonance energy is located in the window of nominal optical transparency of the semiconductor $\hbar \omega <E_{0}$ . To achieve this, the aluminum content in the ${\mathrm{Al}}_{x}$ ${\mathrm{Ga}}_{1-x}$ As solid solution should be ultimately high x≈0.9−1 . In the spectral region of LSPR, the optical properties of such a metamaterial are mostly determined by the embedded system of nanoparticles.

The large oscillator strength of the LSPR makes the resonant value of the optical absorption coefficient as strong as (4–6) $\;\times \;10^{4}$ ${\mathrm{cm}}^{-1}$ . This value is higher than the direct band-to-band absorption coefficient in the semiconductor matrix in the spectral range $E_{0}<\hbar \omega <E_{0}+\Delta_{0}$ . So, the LSPR can be used to enhance the optical absorption of the medium at and above the band gap of the semiconductor. The plasmonic response in a metal–semiconductor metamaterial can potentially improve the performance of photodetectors and various photonic devices [36].

The large spectral width of LSPR allows for the observation of LSPR in the optical extinction spectra in the form of strong tails of photon absorption with energy significantly lower than the resonant one. The magnitude of the LSPR-related tails appears to be stronger compared to the tails due to the dielectric losses in the semiconductor matrix, as well as the absorption due to indirect band-to-band transitions in ${\mathrm{Al}}_{x}$ ${\mathrm{Ga}}_{1-x}$ As with x>0.41 .

In general, the localized surface plasmon resonance in the systems of Sb nanoparticles appears to be an efficient tool with which to modify the optical extinction spectra of ${\mathrm{Al}}_{x}$ ${\mathrm{Ga}}_{1-x}$ As semiconductors. Along with a substantial increase in the optical absorption coefficient, the plasmonic excitations provide an ultrashort relaxation time [7]. Therefore, the metamaterials discussed in this paper can be suitable for the ultrafast processing of the optical signals on the technological platform of the common semiconductor optoelectronics.

## Figures and Tables

**Table 1:** Parameters describing the composition dependence of critical point energies (in eV) $E_{0}$ , $E_{0}+\Delta_{0}$ , $E_{1}$ , and $E_{1}+\Delta_{1}$ .

Parameter	E(GaAs)	E(AlAs)−E(GaAs)	$c_{0}$	$c_{1}$
$E_{0}$ [30]	1.424	1.525	−0.37	0
$E_{0}+\Delta_{0}$ [30]	1.764	1.485	−0.37	0
$E_{1}$ [29]	2.926	0.962	−0.2124	−0.7850
$E_{1}+\Delta_{1}$ [29]	3.170	0.917	−0.0734	−0.9393

## Data Availability

Data are contained within the article.

## Abbreviations

The following abbreviations are used in this manuscript:

LSPR	Localized surface plasmon resonance
MBE	Molecular-beam epitaxy
LT	Low-temperature

## References

[1] Maier S. (2007). Plasmonics: Fundamentals and Applications.

[2] Link S., El-Sayed M.A. (1999). Size and Temperature Dependence of the Plasmon Absorption of Colloidal Gold Nanoparticles. J. Phys. Chem. B.

[3] Schaadt D.M., Feng B., Yu E.T. (2005). Enhanced Semiconductor Optical Absorption via Surface Plasmon Excitation in Metal Nanoparticles. Appl. Phys. Lett..

[4] Suh J.Y., Odom T.W. (2013). Nonlinear Properties of Nanoscale Antennas. Nano Today.

[5] Dyakov S.A., Zhigunov D.M., Marinins A., Shalygina O.A., Vabishchevich P.P., Shcherbakov M.R., Presnov D.E., Fedyanin A.A., Kashkarov P.K., Popov S. (2018). Plasmon induced modification of silicon nanocrystals photoluminescence in presence of gold nanostripes. Sci. Rep..

[6] Babicheva V.E. (2023). Optical Processes behind Plasmonic Applications. Nanomaterials.

[7] Koya A.N., Romanelli M., Kuttruff J., Henriksson N., Stefancu A., Grinblat G., De Andres A., Schnur F., Vanzan M., Marsili M. (2023). Advances in Ultrafast Plasmonics. Appl. Phys. Rev..

[8] Kosobukin V.A. (2022). Two-dimensional Coulomb plasmon-excitons, their spectrum and near-field excitation. Solid State Commun..

[9] Ding F., Bozhevolnyi S.I. (2023). Advances in quantum meta-optics. Materials Today.

[10] Zhao D., Liu Y., Qiu J., Liu X. (2021). Plasmonic Saturable Absorbers. Adv. Photonics Res..

[11] Bogdanov S., Boltasseva A., Shalaev V. (2019). Overcoming Quantum Decoherence with Plasmonics. Science.

[12] West P.R., Ishii S., Naik G.V., Emani N.K., Shalaev V.M., Boltasseva A. (2010). Searching for better plasmonic materials. Laser Photonics Rev..

[13] Toropov N.A., Gladskikh I.A., Gladskikh P.V., Kosarev A.N., Preobrazhenskiĭ V.V., Putyato M.A., Semyagin B.R., Chaldyshev V.V., Vartanyan T.A. (2017). Absorption and Photoluminescence of Epitaxial Quantum Dots in the Near Field of Silver Nanostructures. J. Opt. Technol..

[14] Kosarev A.N., Chaldyshev V.V., Kondikov A.A., Vartanyan T.A., Toropov N.A., Gladskikh I.A., Gladskikh P.V., Akimov I., Bayer M., Preobrazhenskii V.V. (2019). Epitaxial InGaAs Quantum Dots in Al_0.29_Ga_0.71_As Matrix: Intensity and Kinetics of Luminescence in the Near Field of Silver Nanoparticles. Opt. Spectrosc..

[15] Melloch M.R., Woodall J.M., Harmon E.S., Otsuka N., Pollak F.H., Nolte D.D., Feenstra R.M., Lutz M.A. (1995). Low-Temperature Grown III-V Materials. Ann. Rev. Mater. Sci..

[16] Bert N.A., Veinger A.I., Vilisova M.D., Goloshchapov S.I., Ivonin I.V., Kozyrev S.V., Kunitsyn A.E., Lavrent’eva L.G., Lubyshev D.I., Preobrazhenskii V.V. (1993). Gallium Arsenide Grown by Molecular Beam Epitaxy at Low Temperatures: Crystal Structure, Properties, Superconductivity. Phys. Solid State.

[17] Liu X., Prasad A., Nishio J., Weber E.R., Liliental-Weber Z., Walukiewicz W. (1995). Native Point Defects in Low-Temperature-Grown GaAs. Appl. Phys. Lett..

[18] Lavrent’eva L.G., Vilisova M.D., Preobrazhenskii V.V., Chaldyshev V.V. (2002). Low-Temperature Molecular Beam Epitaxy of GaAs: Influence of Crystallization Conditions on Structure and Properties of Layers. Crystallogr. Rep..

[19] Warren A.C., Woodall J.M., Freeouf J.L., Grischkowsky D., McInturff D.T., Melloch M.R., Otsuka N. (1990). Arsenic Precipitates and the Semi-Insulating Properties of GaAs Buffer Layers Grown by Low-Temperature Molecular Beam Epitaxy. Appl. Phys. Lett..

[20] Nolte D.D. (1994). Optical Scattering and Absorption by Metal Nanoclusters in GaAs. J. Appl. Phys..

[21] Ushanov V.I., Chaldyshev V.V., Il’inskaya N.D., Lebedeva N.M., Yagovkina M.A., Preobrazhenskii V.V., Putyato M.A., Semyagin B.R. (2014). Fröhlich Resonance in the AsSb/AlGaAs System. Phys. Solid State.

[22] Ushanov V.I., Chaldyshev V.V., Bert N.A., Nevedomsky V.N., Il’inskaya N.D., Lebedeva N.M., Preobrazhenskii V.V., Putyato M.A., Semyagin B.R. (2015). Plasmon Resonance in New AsSb–AlGaAs Metal–Semiconductor Metamaterials. Semiconductors.

[23] Bert N., Ushanov V., Snigirev L., Kirilenko D., Ulin V., Yagovkina M., Preobrazhenskii V., Putyato M., Semyagin B., Kasatkin I. (2022). Metal–semiconductor AsSb-Al_0.6_Ga_0.4_As_0.97_Sb_0.03_ Metamaterial. Materials.

[24] Snigirev L., Ushanov V., Ivanov A., Bert N., Kirilenko D., Yagovkina M., Preobrazhenskii V., Putyato M., Semyagin B., Kasatkin I. (2023). Structure and Optical Properties of a Composite AsSb-Al_0.6_Ga_0.4_As_0.97_Sb_0.03_ Metamaterial. Semiconductors.

[25] Silkin V.M., Eremeev S.V., Ushanov V.I., Chaldyshev V.V. (2023). Localized Surface Plasmon Resonance in Metamaterials Composed of As_1−*z*_Sb_*z*_ Semimetal Nanoparticles in Al_*x*_Ga_1−*x*_As_1−*y*_Sb_y_ Semiconductor Matrix. Nanomaterials.

[26] Bohren C.F., Huffman D.R. (2008). Absorption and Scattering of Light by Small Particles.

[27] Adachi S. (1985). GaAs, AlAs and Al_*x*_Ga_1−*x*_As: Material Parameters for Use in Research and Device Applications. J. Appl. Phys..

[28] Djurišić A.B., Rakić A.D., Kwok P.C.K., Li E.H., Majewski M.L., Elazar J.M. (1999). Modeling the Optical Constants of Al_*x*_Ga_1−*x*_As Alloys. J. Appl. Phys..

[29] Kim C.C., Garland J.W., Raccah P.M. (1993). Modeling the Optical Dielectric Function of the Alloy System *Al*_*x*_*Ga*_1−*x*_As. Phys. Rev. B.

[30] Ioffe Institute NSM Archive: Band Structure and Carrier Concentration of AlGaAs. https://www.ioffe.ru/SVA/NSM/Semicond/AlGaAs/bandstr.html.

[31] Runge E., Gross E.K.U. (1984). Density-Functional Theory for Time-Dependent Systems. Phys. Rev. Lett..

[32] Petersilka M., Gossmann U.J., Gross E.K.U. (1996). Excitation Energies from Time-Dependent Density-Functional Theory. Phys. Rev. Lett..

[33] Silkin V.M., Chulkov E.V., Sklyadneva I.Y., Panin V.E. (1984). Self-Consistent Calculation of the Electron Energy Spectrum of Aluminum. Sov. Phys. J..

[34] Silkin V.M., Chulkov E.V., Echenique P.M. (2003). First-principles Calculation of the Electron Inelastic Mean Free Path in Be Metal. Phys. Rev. B.

[35] Wang X., Kunc K., Loa I., Schwarz U., Syassen K. (2006). Effect of Pressure on the Raman Modes of Antimony. Phys. Rev. B.

[36] Vora A., Gwamuri J., Pala N., Kulkarni A., Pearce J.M., Güney D.Ö. (2014). Exchanging ohmic losses in metamaterial absorbers with useful optical absorption for photovoltaics. Sci. Rep..

